# ECT2 overexpression promotes the polarization of tumor-associated macrophages in hepatocellular carcinoma via the ECT2/PLK1/PTEN pathway

**DOI:** 10.1038/s41419-021-03450-z

**Published:** 2021-02-08

**Authors:** Dafeng Xu, Yu Wang, Jincai Wu, Zhensheng Zhang, Jiacheng Chen, Mingwei Xie, Rong Tang, Cheng Chen, Liang Chen, Shixun Lin, Xiangxiang Luo, Jinfang Zheng

**Affiliations:** 1 Department of Hepatobiliary and Pancreatic Surgery, Hainan General Hospital, Hainan Affiliated Hospital of Hainan Medical University, Haikou, Hainan China; 2 Geriatric Medicine Center, Hainan General Hospital, Hainan Affiliated Hospital of Hainan Medical University, Haikou, Hainan China

**Keywords:** Cancer genetics, Tumour biomarkers

## Abstract

Hepatocellular carcinoma (HCC) is a common high-mortality cancer, mainly due to diagnostic difficulties during its early clinical stages. In this study, we aimed to identify genes that are important for HCC diagnosis and treatment, and we investigated the underlying mechanism of prognostic differences. Differentially expressed genes (DEGs) were identified by using the limma package, and receiver operating characteristic curve analysis was performed to identify diagnostic markers for HCC. Bioinformatics and clinical specimens were used to assess epithelial cell transforming 2 (*ECT2*) in terms of expression, prognostic value, pathways, and immune correlations. In vitro experiments were used to investigate the underlying mechanism and function of *ECT2*, and the results were confirmed through in vivo experiments. The integrated analysis revealed 53 upregulated DEGs, and one candidate biomarker for diagnosis (*ECT2*) was detected. High expression of *ECT2* was found to be an independent prognostic risk factor for HCC. *ECT2* expression showed a strong correlation with tumor-associated macrophages. We found that *ECT2* overexpression increased the migration and proliferation of HCC cells. It also promoted the expression of PLK1, which subsequently interacted with PTEN and interfered with its nuclear translocation, ultimately enhancing aerobic glycolysis and promoting M2 macrophage polarization. M2 macrophages suppress the functions of NK cells and T cells, and this was confirmed in the in vivo experiments. Overall, *ECT2* may promote the polarization of M2 macrophages by enhancing aerobic glycolysis and suppressing the functions of immune cells. *ECT2* could serve as a candidate diagnostic and prognostic biomarker for HCC.

## Introduction

Hepatocellular carcinoma (HCC) is the fifth most common cancer and has the third highest cancer-related death rate^1,2^. While preferable diagnostic criteria and improved therapy have been applied in recent years, the current standard treatments, such as surgical resection and liver transplantation, cannot produce satisfactory results for patients with advanced stages of HCC^3–5^. Therefore, it is critical to have a cancer biomarker that can be used to diagnose HCC and predict HCC prognosis.

The epithelial cell transformation sequence 2 (ECT2) protein is an exchange factor in the Rho band^6^. The activation of the ECT2/Rho pathway may promote the progression of several tumors^7^. ECT2 may promote the progression of human HCC by regulating the Rho/ERK signaling axis^8–10^. ECT2 also plays a tumor-promoting role in a number of other cancers, such as non-small cell lung cancer and breast cancer^11–14^. However, the underlying mechanism responsible for the function of ECT2 has remained unclear.

Whereas normal cells generate ATP through mitochondrial respiration, tumor cells tend to metabolize glucose to lactate, even in the presence of sufficient oxygen, which is known as the Warburg effect^15,16^. An increase in the Warburg effect facilitates migration and proliferation, allowing tumor cells to resist apoptosis via aerobic processes. Importantly, increased production of lactic acid leads to an acidic tumor microenvironment, and the lower pH of the environment influences immune cells in various ways, such as suppressing the functions of T cells and natural killer (NK) cells and promoting the recruitment of regulatory T cells to drive immune escape^17,18^. Lactic acid production also promotes the polarization of macrophages^19^. Macrophages infiltrating the tumor microenvironment can be divided into activated (M1) and alternatively activated (M2) phenotypes, with M2 macrophages suppressing immune cell activity and promoting tumor progression^20^.

In this study, we explored potential diagnostic and prognostic biomarkers and their biological functions in HCC through an integrated bioinformatics analysis, and we ultimately identified ECT2 as a biomarker. The results of in vitro experiments confirmed that ECT2 overexpression caused PLK1 upregulation and subsequently interfered with the nuclear translocation of PTEN, promoted aerobic glycolysis, increased lactate acid production, and enhanced M2 macrophages activity, which suppressed immune cell function. The proposed mechanism was verified by the results of in vivo experiments, which showed that ECT2 reduced the effect of decreased infiltration of M2 macrophages, promoted the apoptosis of tumor cells, and increased the activity of NK cells in the tumor microenvironment.

## Materials and methods

### Data downloading

The GSE76311, GSE101685, GSE101728, and GSE76427 cohorts and corresponding platform annotation files (GPL files) were downloaded from the Gene Expression Omnibus (GEO) database (http://www.ncbi.nlm.nih.gov/geo/). The GSE76311 cohort contained 61 HCC samples and 58 normal samples; the GSE101685 cohort contained 24 HCC samples and 8 normal samples; the GSE101728 cohort contained 7 HCC samples and 7 normal samples; the GSE76427 cohort, which was used for weighted gene co-expression network analysis (WGCNA) analysis, included 115 HCC samples and 52 normal samples. The gene expression profiles of HCC were downloaded from The Cancer Genome Atlas (TCGA) database (https://www.cancer.gov), including 371 HCC samples and 50 normal samples.

### Identification of differentially expressed genes

The limma R package was used to identify DEGs in 3 cohorts (GSE76311, GSE101685, GSE101728). A log2 fold change (FC) value > 1 and an adjusted *p* value < 0.05 were used to identify upregulated DEGs, while a log2 FC value < –1 and an adjusted *p* value < 0.05 were used to identify downregulated DEGs. The intersections of upregulated DEGs in these three cohorts were taken as common DEGs, and these DEGs were validated in the TCGA-LIHC cohort.

### Identification of hub genes by receiver operating characteristic (ROC) analysis

The pROC R package was used to identify genes for HCC diagnosis in the GSE76311 and TCGA cohorts. A forest plot of the top 20 genes with the largest area under the curve (AUC) was drawn by using the forest plot R package.

### Validation of the effectiveness of the *ECT2* gene in the diagnosis of liver cancer by logistics regression analysis

Using the TCGA cohort, a logistic regression model was established to further evaluate the effectiveness of ECT2 in the diagnosis of HCC. By constructing a fivefold cross-validated ROC curve and confusion matrix of ECT2, the diagnostic value of ECT2 in HCC was analyzed.

### Expression of ECT2 in the Oncomine database

Oncomine was used to analyze the expression of ECT2 in multiple tumors. Oncomine is a classic cancer gene chip database that is often used to identify target molecules worth studying or to predict phenotypes. The screening conditions in this study were: (1) gene: ECT2; (2) threshold value setting conditions: *p* < 1e–4, FC > 2, and gene rank = top 10%.

### Functional and pathway enrichment analyses

The enrichment analysis included four methods: over-representation analysis, functional class scoring, pathway topology analysis, and network topology analysis. Gene set enrichment analysis (GSEA) belonged to the category of functional class scoring, and it was mainly used to study whether the priori gene set showed statistically significant differences in two biological states. Kyoto Encyclopedia of Genes and Genomes (KEGG) is a database used for analyzing gene functions. It links genomic information with gene functions, and it aims to reveal the genetic and chemical blueprints of life phenomena.

The TCGAbiolinks package was used to download TCGA-LIHC transcriptome data and clinical information. Tumor samples were extracted according to the sample barcode. They were divided into high- and low-expression groups according to the median ECT2 expression. The limma package was used for differential analysis. Clusterprofiler was used to perform GSEA. The C5 cohort in the Molecular Signatures Database (MSIGDB) was used as the functional background cohort, and the C2 cohort in MSIGDB was used as the pathway background cohort.

### Weighted gene co-expression network analysis

The WGCNA R package was used for co-expression network construction. This method is used to find co-expressed gene modules through scale-free clustering and dynamic tree cutting analysis of expression profiles, with exploration of the associations between genetic networks and phenotypes. The WGCNA algorithm was used to mine the relevant modules of ECT2 with high and low expression levels, and the correlations between these modules and ECT2 expression levels were analyzed.

### Tumor immune infiltration analysis

The TIMER database (https://cistrome.shinyapps.io/timer/) was used for tumor immune infiltration analysis. This database is used to systematically analyze immune infiltration of different cancer types, including 10,897 samples of 32 cancer types from the TCGA database. This study explored correlations between the expression of the *ECT2* gene and macrophages. In addition, we used the “Diff Exp” function to explore differences in the expression of the *ECT2* gene in pan-cancer and control samples.

### Cox proportional-hazards model analysis

The Cox proportional-hazards model is a semiparametric regression model that was proposed by British statistician D. R. Cox (1972). It is mainly used for the prognostic analysis of tumors and other chronic diseases, and it can be used for etiology exploration in cohort studies. The Cox model can be represented by the hazard function, *h* (*t*). In simple terms, it is the risk of death at time *t*. That is, *h*(*t*) = *h*0(*t*) × exp(*b*1 × 1 + *b*2 × 2 + …+ bp*xp), where *h*0(*t*) represents the baseline risk and exp (*bi*) refers to the hazard ratio (HR), with HR = 1: no effect; HR < 1: reduced risk; and HR > 1: increased risk. After grouping the samples according to the level of ECT2 expression, the coxph function was used to calculate the HR and *p* value in each clinical sub-classification.

### Sample collection

HCC and adjacent tissues were collected from 20 patients, immediately placed in liquid nitrogen, and preserved at –80 °C. The included patients and their families were fully informed, and informed consent was obtained from the participants. The study was approved by the Ethics Committee of Hainan General Hospital, Hainan Affiliated Hospital of Hainan Medical University.

### Immunohistochemical staining

The HCC samples were fixed in 10% formalin, embedded in paraffin, and sliced into 5-μm serial sections. The samples were dewaxed with gradient ethanol (from high to low). The tissue sections were placed in a repair box filled with citric acid antigen repair buffer (pH 6.0) for antigen repair in a microwave oven, and then they were cooled to room temperature. The slices were placed in 3% hydrogen peroxide solution, then incubated at room temperature in the dark for 25 min. The slides were placed in phosphate-buffered saline (PBS) (pH 7.4) and washed with shaking on a decolorizing shaker three times (5 min each time) to block the endogenous peroxidase. Then, blocking was performed by incubation in goat serum at 37 °C for 30 min. The samples were incubated in rabbit anti-ECT2 (Thermo Scientific, PA5-67612, 1:20) at 4 °C overnight. They were incubated with horseradish peroxidase-conjugated goat anti-rabbit secondary antibody and placed at 37 °C for 50 min, then stained with 3,3′-diaminobenzidine. The experimental procedure was performed with strict adherence to the manufacturers’ instructions.

### Measurements of glucose, lactate, and glutamine

Cells were seeded in culture plates and cultured for 48 h after gene overexpression or knockdown. The culture media were collected to measure the levels of glucose, lactate, and glutamine with a glucose assay kit (Eton Bioscience), a lactate assay kit (Eton Bioscience), and a glutamine/glutamate determination kit (Sigma), respectively, and the cell numbers were normalized.

### RNA extraction and real-time polymerase chain reaction (PCR) assay

Total RNA was extracted by using TRIzol Reagent (Invitrogen, CA, USA) following the manufacturer’s protocol, and it was reverse-transcribed into complementary DNA by using the Superscript Reverse Transcriptase Kit (Transgene, France). The SuperMix SYBR Green Kit (Transgene, France) was used to carry out real-time PCR in an ABI7300 real-time PCR system (Applied Biosystems). The ECT2 primers were: forward: TGAAGGCAAAGTGACCTGTGA; reverse: AGGCGTCCAGATAGGAGAGC.

### Antibodies, reagents, and cell lines

Anti-PTEN, PLK1, laminB, GAPDH rabbit polyclonal (Abcam, Cambridge, UK), and anti-actin polyclonal (Santa Cruz Biotechnology) antibodies were used at a dilution of 1:1000 for western blotting. Anti-rabbit polyclonal secondary horseradish peroxidase-conjugated antibodies were used for detection (diluted 1:2000). Paclitaxel was purchased from Sigma (St. Louis, MO, USA). The working stock was diluted in the media at a final concentration of 4 μM and further diluted when needed. The human HCC cancer cells (H22 HHCC and HepG2 cells) were obtained from the American Type Culture Collection (Manassas, VA, USA). These cells were cultured in Dulbecco’s modified Eagle’s medium plus 10% fetal bovine serum at 5% CO_2_ and 37 °C.

### Detection of natural killer cell activity

NK cells were added to 1 × 10^5^ cancer cells, which were labeled with CFSE, and incubated at 37 °C for 5 h. After incubation, all cells were stained with 7-AAD (BD Bioscience). Cells were subsequently analyzed by flow cytometry.

### Flow cytometry analysis

Cells were digested with trypsin and then washed with PBS. The Annexin V-FITC Apoptosis Detection Kit (Beyotime) was used to practice cell apoptosis in line with the manufacturer’s instructions. The apoptotic cells were dual-stained with propidium iodide and Annexin V-FITC, using the Annexin V/FITC kit (Thermo Scientific, Shanghai, China). Analysis was carried out with a BDTM LSRІІ flow cytometer (BD Biosciences). Afterward, the data were measured with Cell Quest software (BD Biosciences, San Jose, CA, USA).

### Cell viability assay

Cell viability was determined by using Cell Counting Kit-8 (CCK8) assays. Briefly, cancer cells were seeded in 96-well plates (5 × 10^3^ cells/well) and treated with corresponding processes. CCK8 was added into the wells for 3 h at indicated times. The absorbance in each well at a wavelength of 450 nm (A450 nm) was measured with a ThermoMax microplate reader.

### Western blot analysis and immunoprecipitation

Cancer cells were collected, washed twice with cold PBS, and lysed in NP-40 lysis buffer for 30 min at 4 °C. Protein concentrations were measured by using a bicinchoninic acid assay kit (Thermo). Protein extracts were separated by electrophoresis in a premade 8–12% sodium dodecyl sulfate-polyacrylamide minigel and transferred to a polyvinylidene difluoride membrane. The membrane was incubated with indicated antibodies and detected by using the chemiluminescence method. For immunoprecipitation, total cell lysates were incubated with appropriate antibodies overnight and subsequently rotated with protein A/G beads for 2–4 h at 4 °C. The beads were washed three times with NP-40 lysis buffer, mixed with 2× SDS sample buffer, and boiled for 5–10 min. The co-precipitates were analyzed by western blot analysis.

### Animal experiments

Animal assays were performed in accordance with Experimental Animal Care Guidelines. BALB/c mice (6–7 weeks old) were bred under specific pathogen-free conditions. The mice were divided into four randomized groups (*n* = 6 per group), and 1 × 10^5^ H22-mCherry and H22-shECT2 were subcutaneously injected into the flank of each mouse. Tumor size was measured by using a digital Vernier caliper every 3 days. Tumor volume was calculated as follows: volume = 1/2 × (width^2^ × length). All mice were euthanized after 18 days, and the tumors were collected and visually examined. Then, each tumor was cut into sections and subjected to collagenase IV (Invitrogen, CA, USA) digestion for 3 h at 37 °C. After digestion, it was passed through a 70-μm mesh (Miltenyi Biotec, Germany) and analyzed by flow cytometry.

### Statistical analysis

All data were presented as the mean ± standard error of the mean. One-way analysis of variance was adopted to analyze the differences among groups by using SPSS 13.0 (SPSS Inc., Chicago, IL, USA). Pair-wise comparisons were also made between groups by using the Student–Newman–Keuls test. *P* values less than 0.05 were considered statistically significant.

## Results

### Identification of differentially expressed genes and hub gene

**Fig. 1:**
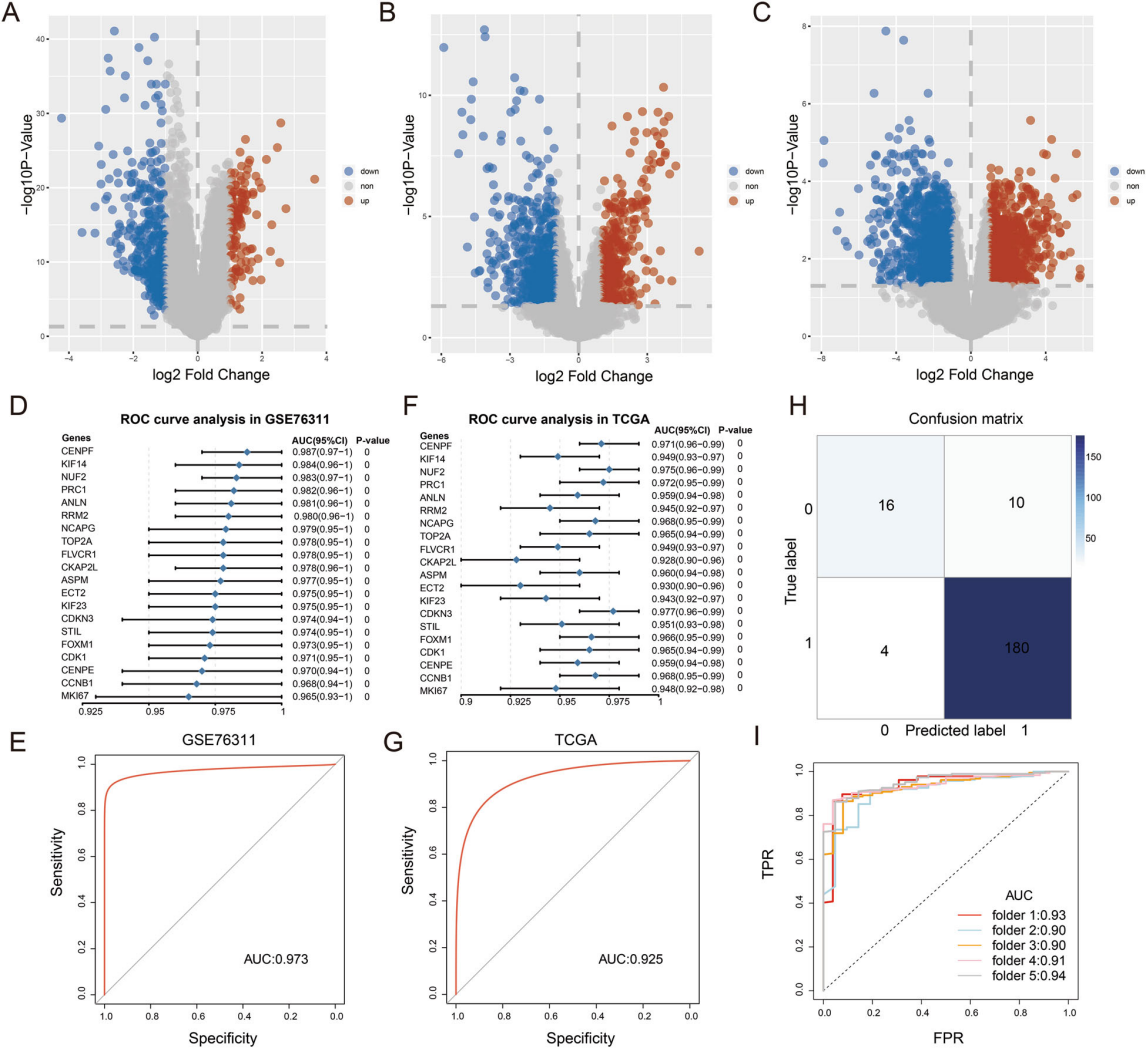
Volcano maps of DEGs and identification of hub genes. **A** Volcano map of DEGs in the GSE76311 cohort. **B** Volcano map of DEGs in the GSE101685 cohort. **C** Volcano map of DEGs in the GSE101728 cohort. **D** Forest map of hub genes in the GSE76311 cohort. **E** ROC curve of the value of ECT2 in predicting diagnosis in the GSE76311 cohort. **F** Forest map of core genes in the TCGA cohort. **G** ROC curve of the value of ECT2 in predicting diagnosis in the TCGA cohort. **H** Confusion matrix of the *ECT2* gene. **I** ROC curve of the fivefold cross-validation of the *ECT2* gene.

The details of the three cohorts used in this study are presented in Supplementary Table 1. Upregulated DEGs were detected in the three cohorts of GSE76311, GSE101685, and GSE101728, with a log2 FC value > 1 and a modified *p* value < 0.05 as the screening criteria. A total of 127 upregulated and 362 downregulated DEGs were obtained from the GSE76311 cohort, 385 upregulated and 601 downregulated DEGs from the GSE101685 cohort, and 913 upregulated and 1255 downregulated DEGs from the GSE101728 cohort (Supplementary Table 2). Figure 1A–C shows the volcano maps of the DEGs in these three cohorts.

The intersections of the 191 DEGs were identified in the three cohorts, including 53 upregulated DEGs and 138 downregulated DEGs (Supplementary Fig. S1A). We then checked the 53 upregulated DEGs by using TCGA database. Supplementary Figure S2B displays the heatmap of the upregulated DEGs.

Functional and pathway enrichment analyses were performed for the upregulated and downregulated DEGs. Supplementary Figure S1C, D shows the upregulated and downregulated DEGs from the functional enrichment analysis. Results from the pathway enrichment analysis of the upregulated and downregulated DEGs are shown in Supplementary Fig. S1E, F. Upregulated DEGs were mainly enriched in Gene Ontology (GO) terms such as mitotic nuclear division and nuclear division, as well as KEGG pathways such as the cell cycle and p53 signaling pathway. Downregulated DEGs were mainly enriched in GO terms such as cellular response to xenobiotic stimulus and response to xenobiotic stimulus, as well as KEGG pathways such as drug metabolism-cytochrome P450 and retinol metabolism.

The upregulated DEGs were used as candidate genes, which were analyzed via ROC curve analysis. The AUC was used to assess the sensitivity and specificity of the candidate genes in the GSE76311 cohort. The top 20 genes were listed based on their AUC values (>0.965), and it was suggested that these genes had the highest diagnostic significance for HCC (Fig. 1D). These genes were *CENPF*, *KIF14*, *NUF2*, *ANLN*, *RRM2*, *NCAPG*, *FLVCR1*, *ASPM*, *CDKN3*, *FOXM1*, *CDK1*, *PRC1*, *TOP2A*, *CKAP2L*, *STIL*, *CENPE*, *CCNB1*, *MKI67*, *KIF23*, and *ECT2*. The AUCs were measured again in The Cancer Genome Atlas Liver Hepatocellular Carcinoma (TCGA-LIHC) cohort to validate the diagnostic significance of these 20 genes (Fig. 1F).

The expression levels and functions of the *ECT2* gene in HCC were not clear, so it was selected as the study’s target gene. The AUCs of ECT2 in terms of predicting diagnosis in the GSE76311 and TCGA-LIHC cohorts were >0.9 (Fig. 1E, G). The TCGA-LIHC cohort was subsequently used to establish a logistic regression model to further assess the effectiveness of ECT2 in determining HCC diagnosis. The results clarified the diagnostic value of ECT2 in HCC (Supplementary Table 3), and we used the results to construct a fivefold cross-validated ROC curve and confusion matrix (Fig. 1H, I).

### Construction of ECT2-based WGCNA network and gene expression and prognosis analysis

**Fig. 2:**
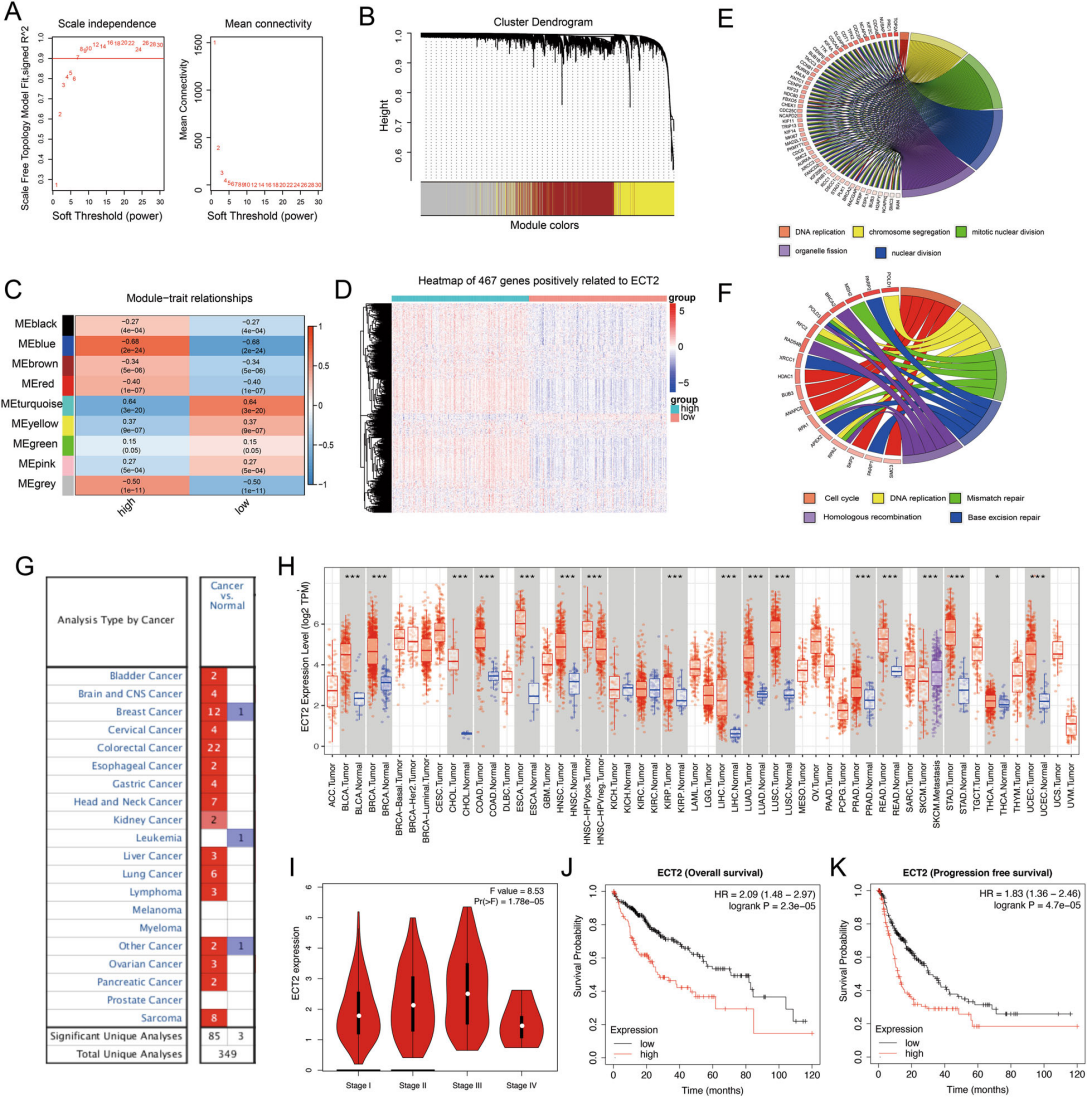
Construction of ECT2-based WGCNA network and gene expression and prognosis analysis. **A** Optimal threshold in the WGCNA. **B** Heatmap of module clustering. **C** Diagram of the module-trait relationships of ECT2. **D** Heatmap of the genes co-expressed with ECT2 in the TCGA-LIHC cohort. **E** GO enrichment analysis of the genes co-expressed with ECT2. **F** KEGG enrichment analysis of the genes co-expressed with ECT2. **G** Expression of ECT2 in multiple tumors in the Oncomine database. Blue indicates low expression in tumor tissue relative to normal tissue, and red indicates high expression in tumor tissue relative to normal tissue. The number represents the number of corresponding studies. **H** Expression of ECT2 in the pan-cancer and control groups. **I** Correlation analysis of ECT2 and clinical stage in the GEPIA database. **J** OS curve of the effect of ECT2 expression in the TCGA-LIHC cohort. **K** PFS curve of the effect of ECT2 expression in the TCGA-LIHC cohort.

The GSE76427 cohort included 115 patients with HCC. The samples were divided into high- and low-expression groups according to ECT2 expression, and WGCNA was used to find modules related to ECT2 expression. The blue modules were most closely related to ECT2 expression, and ECT2 was the hub gene of the blue modules (Fig. 2A–C). The potential biological roles of ECT2 in HCC were further explored. Based on the topological overlap in the WGCNA, 467 genes positively related to ECT2 were identified, and heatmap of gene expression profiles was plotted in the TCGA-LIHC cohort (Fig. 2D).

The GO and KEGG analysis results showed that the genes co-expressed with ECT2 in the hub modules were mainly enriched in GO terms such as replication of DNA and chromosome separation, as well as KEGG pathways such as the cell cycle and mismatch repair (Fig. 2E, F).

The Oncomine database revealed high levels of ECT2 in HCC, prostate cancer, breast cancer, colon cancer, head and neck cancer, lung cancer, and stomach cancer (Fig. 2G). We also used the TIMER database to analyze the expression levels of ECT2 in pan-cancer, which were consistent with those found by using Oncomine, with ECT2 expression being significantly higher in most cancers compared to the control group (Fig. 2H).

Univariate and multivariate Cox analyses were performed on ECT2 expression in the TCGA-LIHC cohort (Supplementary Table 4). Highly expressed ECT2 and Stage III/IV were independent risk factors for overall survival (OS) and progression-free survival (PFS) in HCC. We also found that the expression of ECT2 was positively correlated with the advance of Stages I, II, and III by using the Gene Expression Profiling Interactive Analysis (GEPIA) database (*p* = 1.78e–05) (Fig. 2I). We further plotted survival curves of ECT2 expression in terms of OS and PFS by using the Kaplan–Meier Plotter database. The results showed that the prognosis of patients with high ECT2 expression was worse than that of patients with low expression in terms of OS and PFS (Fig. 2J, K).

### Downregulation of ECT2 inhibits the proliferation and migration of HCC **cells**

**Fig. 3:**
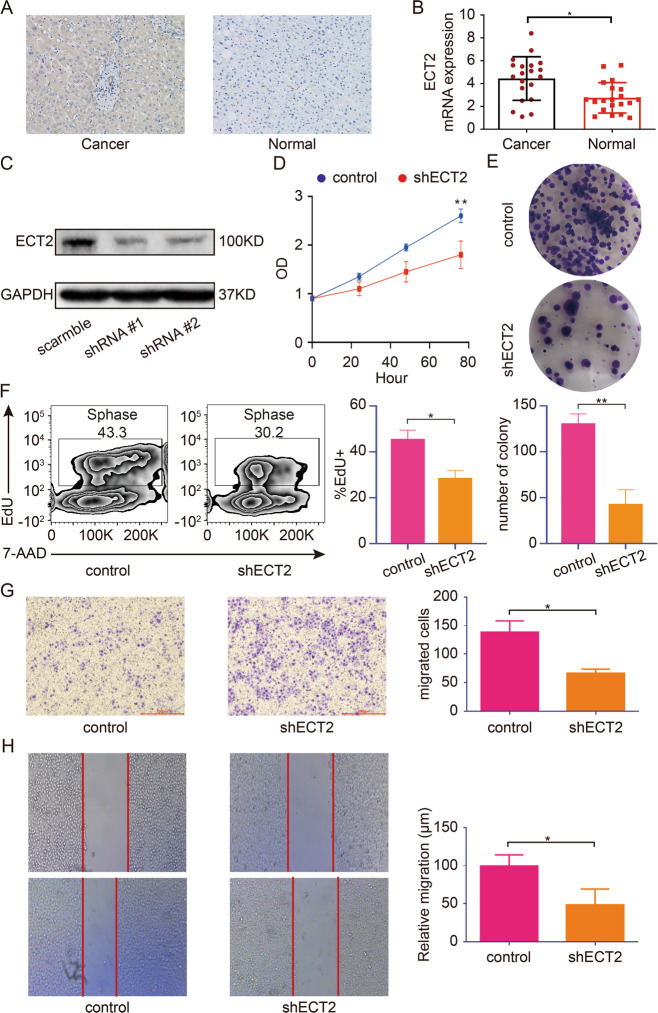
Downregulation of ECT2 inhibits the proliferation and migration of HCC cells. **A** Representative immunohistochemistry stain of ECT2 in HCC and para-cancerous normal tissues. **B** Quantification of PCR analysis of ECT2 expression in HCC and para-cancerous normal tissues. **C** Western blot analysis of ECT2 after transfection of ECT2-targeting shRNA. **D** ECT2 downregulation inhibits cell proliferation. Cancer cells were transfected with ECT2-targeting shRNA, and the absorption (A450 nm) was detected at 0, 24, 48, and 72 h. **E** Cancer cells transfected with ECT2-targeting shRNA and the normal control were assayed for clonogenicity in adherent cultures. **F** EdU incorporation assay was used to examine the proliferation of cancer cells after ECT2 knockdown. **G** Transwell assay was used for cancer cells transfected with ECT2-targeting shRNA (magnification 100×). **H** Wound-healing assay was used for cancer cells transfected with ECT2-targeting shRNA (magnification 50×, scale bar: 500 μm). Error bars represent means ± SD. *p < 0.05, **p < 0.01. NS means “not significant” by paired two-sided Student’s *t*-test.

To confirm the association between ECT2 expression and clinical prognosis, ECT2 expression levels in 20 clinical samples were analyzed. HCC tissue was found to express higher ECT2 compared with para-cancerous normal tissue (Fig. 3A). PCR analysis was used to confirm this conclusion. HCC tissue had higher ECT2 transcription activity compared with para-cancerous normal tissue (Fig. 3B). Taken together, these results suggested that ECT2 was overexpressed in HCC tissue compared with normal tissue. ECT2 expression decreased significantly after the transfection of shRNA targeting ECT2, suggesting that ECT2 was successfully knocked down in HCC cells (Fig. 3C). CCK8 and colony genesis assays were used to measure the effect of ECT2 expression on proliferation. ECT2 downregulation inhibited the proliferation of HCC cells (Fig. 3D, E). To further support this assumption, 5-ethynyl-2’-deoxyuridine assay was conducted, and the S phase decreased after ECT2 was knocked down (Fig. 3F). To evaluate the effect of ECT2 expression on the migration of HCC cells, wound-healing and transwell migration assays were carried out. The migration of HCC cells was significantly inhibited after ECT2 was knocked down (Fig. 3G, H). Taken together, these results suggested that HCC cell migration and proliferation were impaired by ECT2 downregulation.

### ECT2 overexpression promotes AKT activation via the PLK1/PTEN pathway

**Fig. 4:**
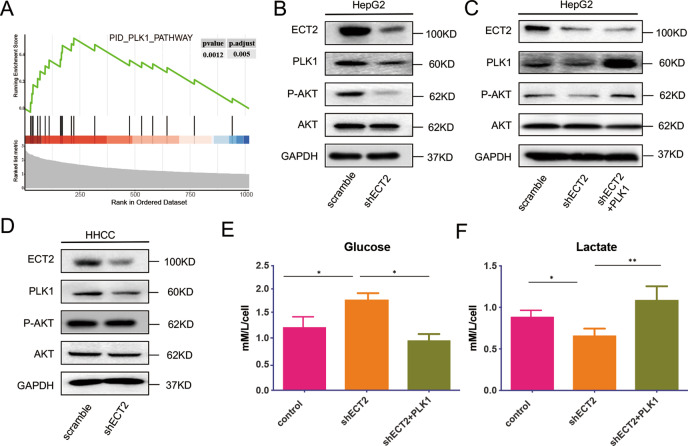
ECT2 overexpression promotes activation of AKT via the PLK1/PTEN pathway. **A** Enrichment analysis of ECT2 pathway functions in the TCGA-LIHC cohort. **B**, **C** Western blot analysis of p-AKT, PLK1, and AKT expression levels after ECT2 knockdown or PLK1 upregulation in the HepG2 cell line. **D** Western blot analysis of p-AKT, PLK1, and AKT expression levels after ECT2 knockdown in the PTEN-missing cell line of HCC. **E** Glucose intake by cancer cells was evaluated after ECT2 knockdown. **F** Lactate acid production by cancer cells was evaluated after ECT2 knockdown. Error bars represent means ± SD. *p < 0.05, **p < 0.01. NS means “not significant” by paired two-sided Student’s *t*-test.

GSEA results showed that ECT2 was significantly enriched in pathways related to the PID_PLK1 pathway (Fig. 4A).

Western blot analysis was performed to evaluate the downstream signals affected by ECT2 knockdown (Fig. 4B). After ECT2 was downregulated, the expression of PLK1 was significantly inhibited, followed by impaired phosphorylation of AKT. After overexpression of PLK1, the decreased AKT phosphorylation was partially improved, suggesting that the expression of ECT2 affected AKT phosphorylation in a PLK1-dependent manner (Fig. 4C). PLK1 has been reported to influence AKT activation in a PTEN-dependent manner. To confirm this assumption, the HHCC cell line, which is an HCC cell line that is missing PTEN, was used to investigate the role of PTEN in ECT2 function. No significant activation of AKT was observed after ECT2 was knocked down in the HHCC cell line (Fig. 4D). This suggested that ECT2 regulated AKT in a PTEN-dependent manner. PTEN is an important molecule that regulates metabolism in cancer cells. As expected, glucose intake was increased after ECT2 was downregulated (Fig. 4E), and it was partially rescued after PLK1 was overexpressed. Similarly, lactate acid production decreased after ECT2 was downregulated, and it increased after PLK1 was overexpressed (Fig. 4F).

### PLK1 interacts with PTEN and inhibits PTEN nuclear translocation

**Fig. 5:**
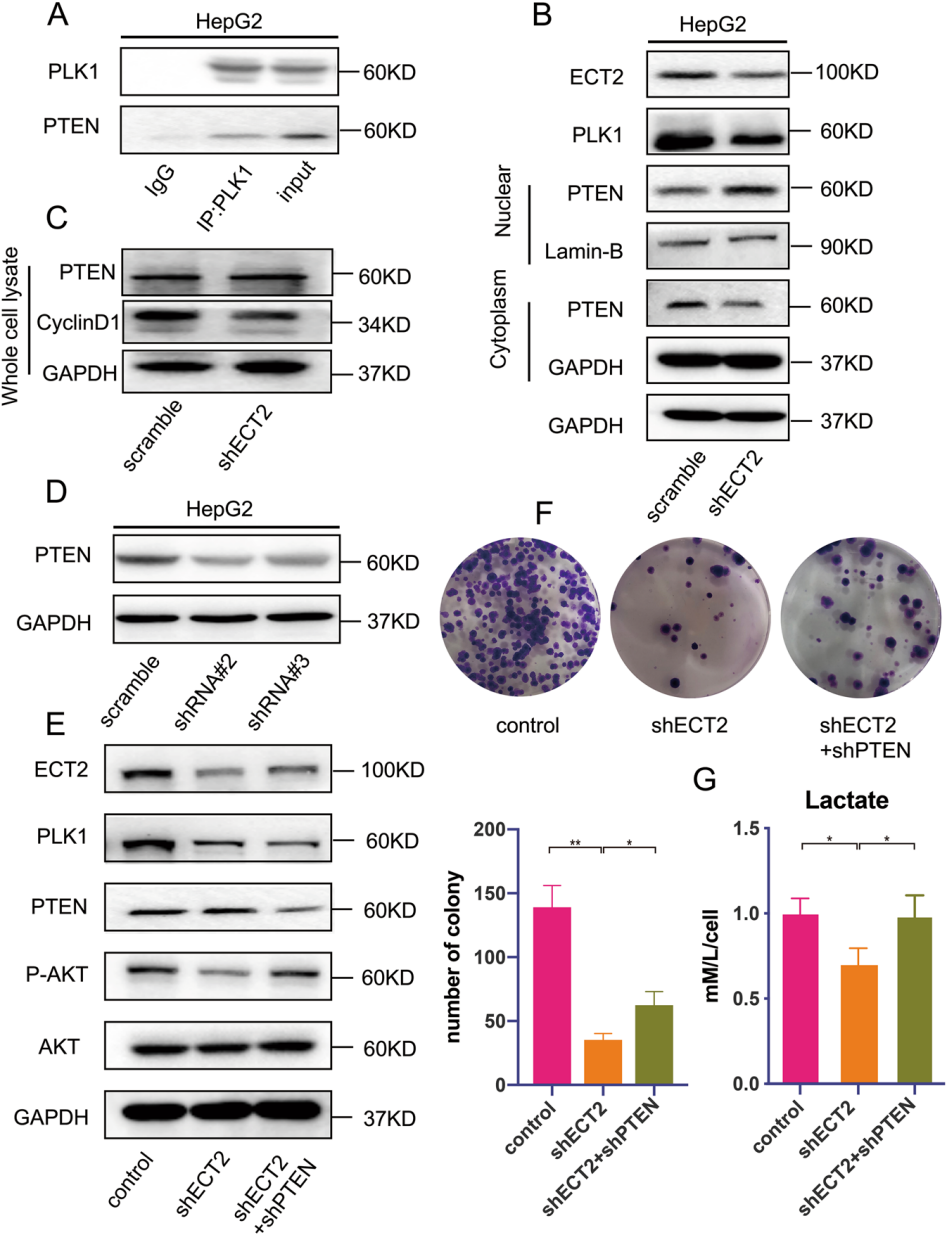
PLK1 interacts with PTEN and inhibits PTEN nuclear translocation. **A** Co-immunoprecipitation analysis with anti-PLK1 antibody or IgG in HepG2 cells. **B** Nuclear translocation of PTEN after ECT2 downregulation was evaluated by western blot analysis. **C** Western blot analysis was used to confirm cyclin D1 and PTEN expression levels in whole-cell lysate after ECT2 was downregulated. **D** Western blot analysis was used to evaluate the effect of PTEN knockdown. **E** Western blot analysis of p-AKT, PLK1, and AKT expression levels after ECT2 or PTEN knockdown in the HepG2 cell line. **F** Cancer cells transfected with ECT2- and PTEN-targeting shRNA were assayed for clonogenicity in adherent cultures. **G** Lactate acid production by cancer cells was evaluated after ECT2 knockdown. Error bars represent means ± SD. *p < 0.05, **p < 0.01. NS means “not significant” by paired two-sided Student’s *t*-test.

To confirm the underlying role of PTEN in the ECT2 pathway, co-immunoprecipitation analysis was performed. PLK1 was found to precipitate with PTEN, suggesting that PLK1 may directly interact with PTEN (Fig. 5A). After ECT2 was downregulated, PLK1 expression was inhibited, and PTEN nuclear translocation increased (Fig. 5B). Taken together, these results suggested that PLK1, which is a downstream signaling molecule of ECT2, could interact with PTEN and subsequently impair the nuclear translocation of PTEN. To assess whether downregulation of ECT2 mainly influenced the nuclear translocation of PTEN rather than its expression, PTEN expression in whole-cell lysate was evaluated. No significant difference in PTEN expression was observed after ECT2 was knocked down (Fig. 5C). It has been reported that the localization of PTEN results in downregulation of cyclin D1. To confirm if the downstream signal of PTEN was influenced by ECT2-induced nuclear localization of PTEN, cyclin D1 expression after ECT2 knockdown was evaluated (Fig. 5C). Cyclin D1 expression decreased as ECT2 was downregulated.

To confirm whether ECT2 influenced the function of HCC cells in a PTEN-dependent pathway, PTEN was knocked down in an ECT2 knockdown cell line (Fig. 5D). After PTEN was knocked down, the reduced AKT activation caused by ECT2 downregulation was partly rescued (Fig. 5E). The impaired proliferation of HCC cells induced by ECT2 knockdown was partially rescued after PTEN downregulation (Fig. 5F). Taken together, these results suggested that PLK1 interacted with PTEN and inhibited PTEN nuclear translocation, and the metabolism and proliferation function of ECT2 was dependent on PTEN expression.

### ECT2 overexpression increases the production of lactic acid and subsequently promotes M2 macrophage polarization

**Fig. 6:**
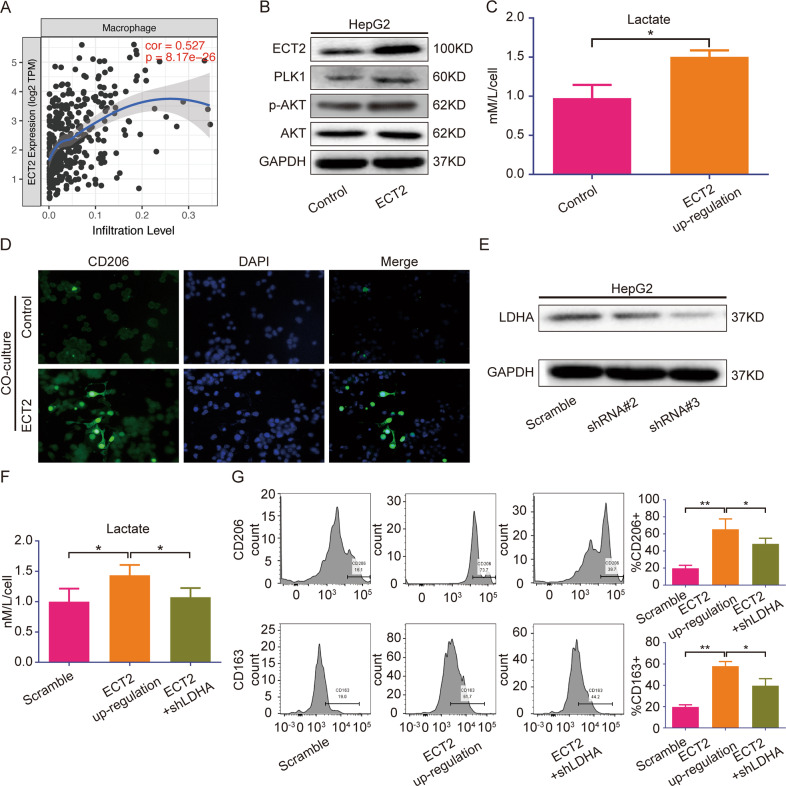
ECT2 overexpression increases the production of lactic acid and subsequently promotes M2 macrophage polarization. **A** Correlation analysis between ECT2 and macrophages. **B** Western blot analysis of p-AKT, PLK1, and AKT expression levels after ECT2 was overexpressed in the HepG2 cell line. **C** Lactate acid production by cancer cells was evaluated after ECT2 was overexpressed. **D** Immunofluorescence analysis of macrophage polarization markers. **E** Western blot analysis was used to evaluate the effect of LDHA knockdown. **F** Lactate acid production by cancer cells was evaluated after ECT2 was overexpressed and/or LDHA was knocked down. **G** CD206 and CD163, which are polarization markers, were evaluated by flow cytometry after macrophages were co-cultured with cancer cells whose LDHA and/or ECT2 expression was influenced. Error bars represent means ± SD. *p < 0.05, **p < 0.01. NS means “not significant” by paired two-sided Student’s *t*-test.

The TIMER database was used to investigate the impact of ECT2 expression on tumor immune infiltration. It was found that the expression of ECT2 was positively correlated with macrophage infiltration (cor = 0.527, *p* = 8.17e–26) (Fig. 6A). It has been reported that tumor cell-generated lactate acid may promote the polarization of M2 macrophages. To investigate this function, ECT2 was overexpressed in HCC cells (Fig. 6B). PLK1 expression was increased as ECT2 was overexpressed, and AKT activation was also enhanced, as expected. The amount of lactate acid produced by tumor cells was significantly enhanced after ECT2 overexpression (Fig. 6C).

CD206, which is a classical marker of M2 macrophage polarization, was increased in macrophages co-cultured with ECT2-overexpressing HCC cells compared with macrophages co-cultured with normal HCC cells (Fig. 6D). To confirm that the lactate acid resulted in increased M2 polarization, lactate dehydrogenase A (LDHA), which is an important enzyme involved in lactate acid production, was knocked down (Fig. 6E). Increased lactate acid production caused by ECT2 overexpression was partly inhibited as LDHA was knocked down (Fig. 6F). We repeated the previous experiments (Fig. 6G). Co-culture with ECT2-overexpressing HCC cells promoted macrophage polarization, but this effect was impaired as LDHA was knocked down.

### ECT2 overexpression-induced macrophages impair immune cell function

**Fig. 7:**
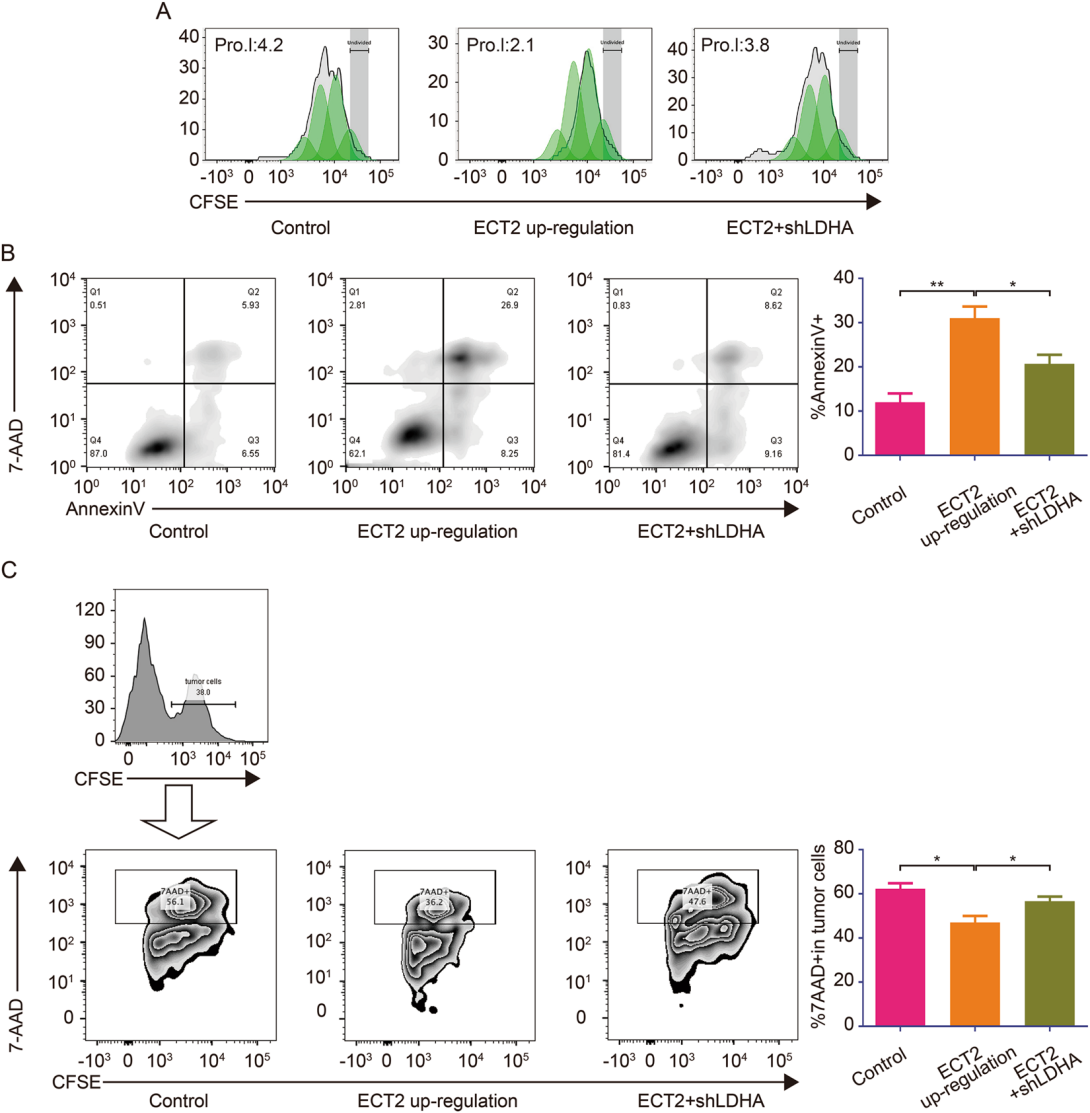
ECT2 overexpression-induced macrophages impair immune cell function. **A** CFSE was used to evaluate the proliferation of T cells. T cells were cultured directly with macrophages, which were induced by cancer cells for 48 h. **B** T cells were cultured directly with macrophages, which were induced by cancer cells for 48 h. The apoptosis rate was evaluated by using Annexin V staining. **C** NK cell cytotoxicity assay was performed. NK cells were pre-co-cultured with macrophages, which were induced by cancer cells. HepG2 cells were used as target cells. The right panel shows the quantification of NK cell cytotoxicity. Error bars represent means ± SD. *p < 0.05, **p < 0.01. NS means “not significant” by paired two-sided Student’s *t*-test.

M2 macrophages have been reported to suppress immune cell function in the tumor microenvironment. To confirm this effect, ECT2 overexpression-induced macrophages were co-cultivated with T cells. Impaired T cell proliferation and enhanced apoptosis were observed, but this effect was inhibited after LDHA was suppressed in the HCC cells (Fig. 7A, B). NK cells are important immune cells in the tumor microenvironment. They can lyse tumor cells without activation beforehand. Following co-culture with cells overexpressing ECT2, the function of NK cells was impaired, but this effect was inhibited after LDHA was downregulated (Fig. 7C).

Taken together, these results suggested that ECT2 promoted lactate acid-dependent polarization of M2 macrophages and subsequently inhibited immune cell activity.

### Downregulation of ECT2 inhibits M2 macrophage infiltration in vivo

**Fig. 8:**
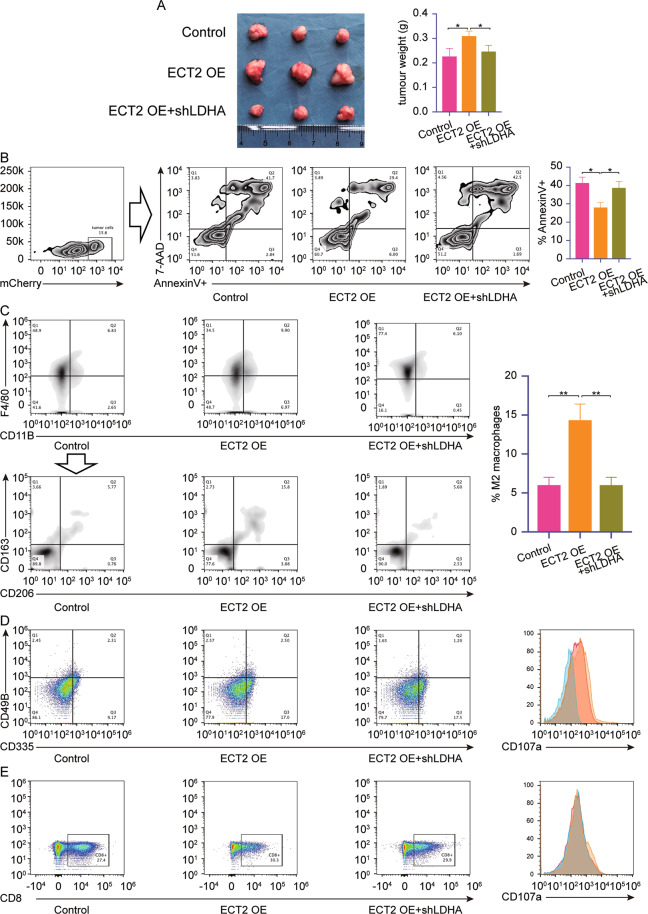
Downregulation of ECT2 inhibits M2 macrophage infiltration in vivo. **A** Weight of tumors isolated from mice with H22 injections (*n* = 3). **B** Apoptosis of tumor cells gated as mCherry+. **C** M2 macrophage infiltration was evaluated by flow cytometry. **D** NK cell infiltration was evaluated. CD107a expression is shown in the right panel. Red: control group, orange: ECT2 OE+shLDHA group, blue: ECT2 OE group. **E** T cell infiltration was evaluated. CD107a expression is shown in the right panel. Red: control group, orange: ECT2 OE+shLDHA group, blue: ECT2 OE group. Error bars represent means ± SD. *p < 0.05, **p < 0.01. N means “not significant” by paired two-sided Student’s *t*-test.

To confirm our findings, animal experiments were performed to investigate the role played by ECT2 in vivo. H22-mCherry, H22-mCherry-ECT2 OE, and H22-ECT2 OE+shLDHA-mCherry were injected into the flanks of BALB/c mice. The mice were euthanized after 3 weeks (Fig. 8A). The tumors produced by H22-mCherry were significantly smaller than those produced by H22-mCherry-ECT2 OE and H22-ECT2 OE+shLDHA-mCherry. This result suggested that ECT2 overexpression promoted proliferation in vivo, but LDHA knockdown impaired this effect.

To investigate the underlying mechanism that caused the tumor volume disparity, single-cell suspensions of subcutaneous tumor cells were analyzed by flow cytometry. Tumor cells in subcutaneous tumors were gated as mCherry+ (Fig. 8B). Compared with normal tumor cells, less apoptosis was observed in the tumor cells with ECT2 overexpression. LDHA knockdown significantly reversed the anti-apoptosis effect of ECT2 overexpression. Macrophage infiltration was confirmed (Fig. 8C), with macrophages gated as F4/80+CD11b+. No significant differences in macrophage infiltration were observed among the groups. The infiltration of M2 macrophages in tumor cells was further analyzed. The M2 macrophages were gated as CD163+CD206+. ECT2 overexpression promoted M2 macrophage infiltration in vivo, but LDHA downregulation impaired this effect. Based on our previous findings, we investigated the effect of ECT2 on immune cell function. NK cells were gated as CD335+CD49B+, and cytotoxic T cells were gated as CD8+cells (Fig. 8D, E). ECT2 expression and LDHA knockdown did not influence the infiltration of NK cells or T cells, but the activation of NK cells, which was indicated as CD107a+, was impaired as ECT2 was overexpressed, and knocking down LDHA reversed this effect. No significant differences were observed in T cell activation between the groups.

Taken together, these results suggested that ECT2 promoted the infiltration of M2 macrophages and suppressed the function of NK cells in vivo.

## Discussion

HCC is a highly aggressive primary liver malignancy and the world’s third leading cause of cancer-related deaths^21–23^. In HCC, the tumor stroma consists of inflammatory cells, fibroblasts, and endothelial cells, which crosstalk and modify the properties of tumor cells, promote tumor progression, and participate in tumor development^24–26^. Tumor-associated macrophages (TAMs) that promote cancer initiation and malignant progression have been identified. Infiltration of TAMs is linked to poor prognosis in lung cancer, thyroid cancer, prostate cancer, and HCC^27–31^, but the underlying control mechanism of the polarization and infiltration of TAMs remains obscure. It has recently been reported that lactic acid, which is a by-product of aerobic glycolysis, may contribute to the polarization of TAMs^19^. Increased production of lactic acid is a hallmark of the Warburg effect. The Warburg effect mainly occurs in tumor cells, in contrast to normal cells, even in the presence of sufficient oxygen, and during this process, pyruvic acid tends to be catalyzed by LDHA into lactic acid^32–34^.

In this study, DEGs were identified in three cohorts of HCC, and ROC curves were used to evaluate the predictive diagnostic performance of the DEGs. The *ECT2* gene was selected as the hub gene for further analysis. The logistic regression model was used to evaluate the high efficacy of ECT2 in the diagnosis of HCC. The results of univariate and multivariate Cox analyses showed that high expression of ECT2 was an independent prognostic risk factor for HCC. GSEA results showed that ECT2 was significantly associated with pathways linked to liver cancer-related pathways and the PID_PLK1 pathway. The TIMER database was used to investigate interactions between the immune infiltration of tumors and ECT2. The correlation between ECT2 and TAMs was the strongest among five other immune cells (cor = 0.527, *p* = 8.17e–26).

The results of the in vitro experiments confirmed that ECT2 knockdown reduced PLK1 expression and subsequently promoted the nuclear translocation of PTEN in HCC cells. ECT2 overexpression leads to a significant Warburg effect and increased lactic acid production. Based on our knowledge, we were curious about whether enhanced lactic acid production could promote TAM polarization. We found that after ECT2 was overexpressed, co-culture with macrophages resulted in enhanced M2 marker expression, suggesting that ECT2 overexpression promoted M2 macrophage polarization. TAMs have been reported to promote immune suppression by suppressing the anti-tumor function of immune cells, such as T cells and NK cells^35,36^. To confirm this theory, ECT2-overexpressing tumor cell-educated macrophages were co-cultured with T cells and NK cells. A significant immune suppression effect was observed in the TAMs that were co-cultured with ECT2-overexpressing tumor cells compared with those that were co-cultured with normal tumor cells. Some of our data showed that ECT2 overexpression promoted the production of lactic acid and subsequently the polarization of M2 macrophages, and polarized macrophages were found to contribute to immune suppression in the HCC microenvironment.

This result was confirmed through in vivo experiments. As expected, increased TAM infiltration was observed in the normal HCC group compared to the ECT2 knockdown group, and ECT2 downregulation promoted tumor cell apoptosis and generated smaller-volume tumors. The infiltration of T cells and NK cells was also detected; no significant differences were observed between the experimental groups, but the function of NK cells was partly rescued after ECT2 was knocked down.

In conclusion, we identified ECT2 as a cancer biomarker that can be used for diagnosis and prognostic prediction in HCC. ECT2 overexpression was found to promote lactic acid production through the ECT2/PLK1/PTEN pathway, enhance TAM infiltration, and suppress immune cell function. Therefore, suppressing ECT2 may be a strategy for HCC prevention and treatment.

Our work has some limitations. The expression of PI (3,4,5) P3 was not evaluated after the nuclear translocation of PTEN, and the mechanism of ECT2 requires further investigation.

Taken together, our results showed that ECT2 overexpression promoted TAM polarization in HCC via the ECT2/PLK1/PTEN pathway.

## Supplementary information

Information of the data sets in this study

Information of differentially expressed genes

Information of five-fold cross-validation

Univariate and multivariate cox analysis

Functional enrichment analysis of DEGs

## Data Availability

The data used to support the findings of this study are available from the corresponding author on reasonable request.

## Code availability

All of the R codes used in this study can be obtained from the corresponding author. The R software used was version 3.60.
