# Correction to: ECT2 overexpression promotes the polarization of tumor-associated macrophages in hepatocellular carcinoma via the ECT2/PLK1/PTEN pathway

**DOI:** 10.1038/s41419-022-04631-0

**Published:** 2022-03-02

**Authors:** Dafeng Xu, Yu Wang, Jincai Wu, Zhensheng Zhang, Jiacheng Chen, Mingwei Xie, Rong Tang, Cheng Chen, Liang Chen, Shixun Lin, Xiangxiang Luo, Jinfang Zheng

**Affiliations:** 1 Department of Hepatobiliary and Pancreatic Surgery, Hainan General Hospital, Hainan Affiliated Hospital of Hainan Medical University, Haikou, Hainan China; 2 Geriatric Medicine Center, Hainan General Hospital, Hainan Affiliated Hospital of Hainan Medical University, Haikou, Hainan China

**Keywords:** Cancer genetics, Tumour biomarkers

Correction to: *Cell Death and Disease* 10.1038/s41419-021-03450-z, published online 0 8 February 2021

During the process of checking the manuscript, the authors found that GAPDH of Figure 4B, Figure 4C; GAPDH of Figure 6B and Figure 6E were incorrectly placed. The P-AKT of Figure 4 D and Figure 6B were incorrectly placed, and the authors would like this to be corrected to prevent unnecessary misunderstandings.

In addition, the label group of statistic analysis shown in Figure 8C was wrongly placed.

The authors apologise for any inconvenience caused and have confirmed that the associated quantitation is correct and that the results of the study were not affected.

The corrected figures can be found below. The original article has been corrected.

**Figure 1:**
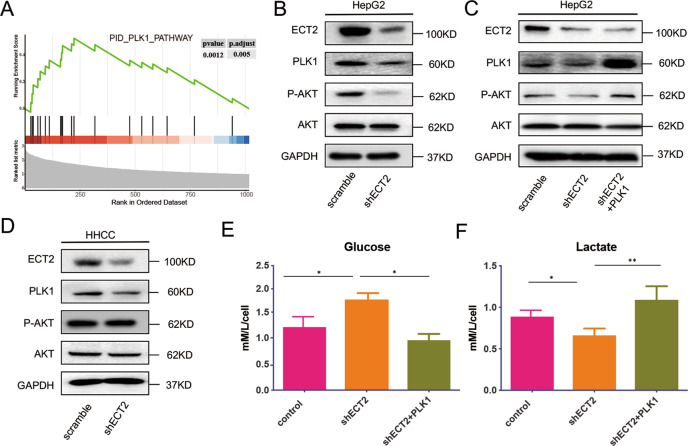
Fig. 4

**Figure 2:**
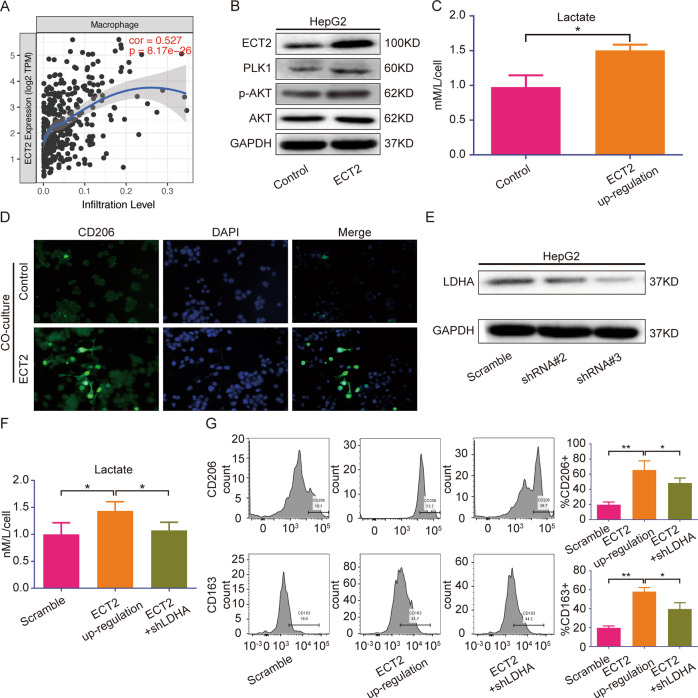
Fig. 6

**Figure 3:**
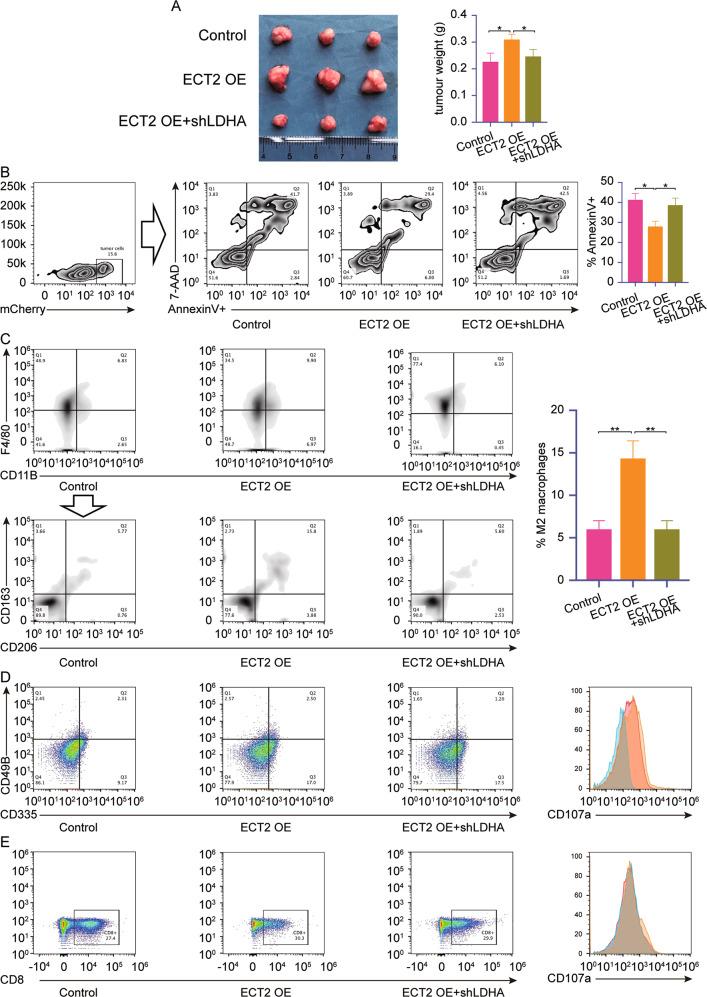
Fig. 8

