# Supplementary material for: ECT2 overexpression promotes the polarization of tumor-associated macrophages in hepatocellular carcinoma via the ECT2/PLK1/PTEN pathway
Source: Cell Death Dis. 2021 Feb 8;12(2):162. doi: 10.1038/s41419-021-03450-z (PMC7870664; doi:10.1038/s41419-021-03450-z)
Supplement: Supplementary file 1 — Information of the data sets in this study [file 41419_2021_3450_MOESM1_ESM.docx]

**S_Table 1: Information of the data sets in this study.**

| Cohorts | Cancer | Normal |
| --- | --- | --- |
| GSE76311 | 61 | 58 |
| GSE101685 | 24 | 8 |
| GSE101728 | 7 | 7 |
| GSE76427 | 115 | 52 |
| TCGA | 371 | 50 |
