# Supplementary material for: ECT2 overexpression promotes the polarization of tumor-associated macrophages in hepatocellular carcinoma via the ECT2/PLK1/PTEN pathway
Source: Cell Death Dis. 2021 Feb 8;12(2):162. doi: 10.1038/s41419-021-03450-z (PMC7870664; doi:10.1038/s41419-021-03450-z)
Supplement: Supplementary file 2 — Information of differentially expressed genes [file 41419_2021_3450_MOESM2_ESM.docx]

**S_Table2. Information of differentially expressed genes**

| Cohorts | Up | down | all |
| --- | --- | --- | --- |
| GSE76311 | 127 | 362 | 489 |
| GSE101685 | 385 | 601 | 986 |
| GSE101728 | 913 | 1255 | 2168 |
