# Supplementary material for: ECT2 overexpression promotes the polarization of tumor-associated macrophages in hepatocellular carcinoma via the ECT2/PLK1/PTEN pathway
Source: Cell Death Dis. 2021 Feb 8;12(2):162. doi: 10.1038/s41419-021-03450-z (PMC7870664; doi:10.1038/s41419-021-03450-z)
Supplement: Supplementary file 3 — Information of five-fold cross-validation [file 41419_2021_3450_MOESM3_ESM.docx]

**S_Table 3: Information of five-fold cross-validation**

|  | Accuracy | Precision | Recall | F1_score |
| --- | --- | --- | --- | --- |
| Sub-folder 1 | 0.93 | 0.95 | 0.98 | 0.96 |
| Sub-folder 2 | 0.90 | 0.95 | 0.93 | 0.94 |
| Sub-folder 3 | 0.9 | 0.94 | 0.95 | 0.94 |
| Sub-folder 4 | 0.91 | 0.93 | 0.97 | 0.95 |
| Sub-folder 5 | 0.94 | 0.95 | 0.98 | 0.97 |
| Average | 0.916 | 0.944 | 0.956 | 0.952 |
