# Supplementary material for: ECT2 overexpression promotes the polarization of tumor-associated macrophages in hepatocellular carcinoma via the ECT2/PLK1/PTEN pathway
Source: Cell Death Dis. 2021 Feb 8;12(2):162. doi: 10.1038/s41419-021-03450-z (PMC7870664; doi:10.1038/s41419-021-03450-z)
Supplement: Supplementary file 4 — Univariate and multivariate cox analysis [file 41419_2021_3450_MOESM4_ESM.docx]

| Parameters | Univariate analysis | | | | Multivariate analysis | | | |
| --- | --- | --- | --- | --- | --- | --- | --- | --- |
| LIHC_OS | HR | lower .95 | upper .95 | p.value | HR | lower .95 | upper .95 | p.value |
| ECT2 expression High vs Low | 2 | 1.4 | 3 | 2.00E-04 | 1.8 | 1.2 | 2.7 | 2.50E-03 |
| gender male vs female | 0.78 | 0.54 | 1.1 | 0.2 | 0.9 | 0.61 | 1.3 | 0.59 |
| tumor_stage III/IV vs I/II | 2.4 | 1.7 | 3.5 | 2.30E-06 | 2.2 | 1.5 | 3.2 | 6.80E-05 |
| age >65 vs <=65 | 1.3 | 0.87 | 1.8 | 0.23 | 1.3 | 0.91 | 1.9 | 0.15 |
| LIHC_PFS | HR | lower .95 | upper .95 | p.value | HR | lower .95 | upper .95 | p.value |
| ECT2 expression High vs Low | 1.7 | 1.3 | 2.2 | 2.40E-04 | 1.5 | 1.2 | 2.1 | 0.0023 |
| gender male vs female | 0.86 | 0.65 | 1.1 | 0.3 | 0.94 | 0.7 | 1.3 | 0.66 |
| tumor_stage III/IV vs I/II | 2 | 1.5 | 2.7 | 2.30E-06 | 1.9 | 1.4 | 2.5 | 3.00E-05 |
| age >65 vs <=65 | 1 | 0.76 | 1.3 | 0.95 | 1.1 | 0.84 | 1.5 | 0.45 |

**S_Table 4．Univariate and multivariate cox analysis**
