# Supplementary material for: ECT2 overexpression promotes the polarization of tumor-associated macrophages in hepatocellular carcinoma via the ECT2/PLK1/PTEN pathway
Source: Cell Death Dis. 2021 Feb 8;12(2):162. doi: 10.1038/s41419-021-03450-z (PMC7870664; doi:10.1038/s41419-021-03450-z)
Supplement: Supplementary file 5 — Functional enrichment analysis of DEGs [file 41419_2021_3450_MOESM5_ESM.pdf]

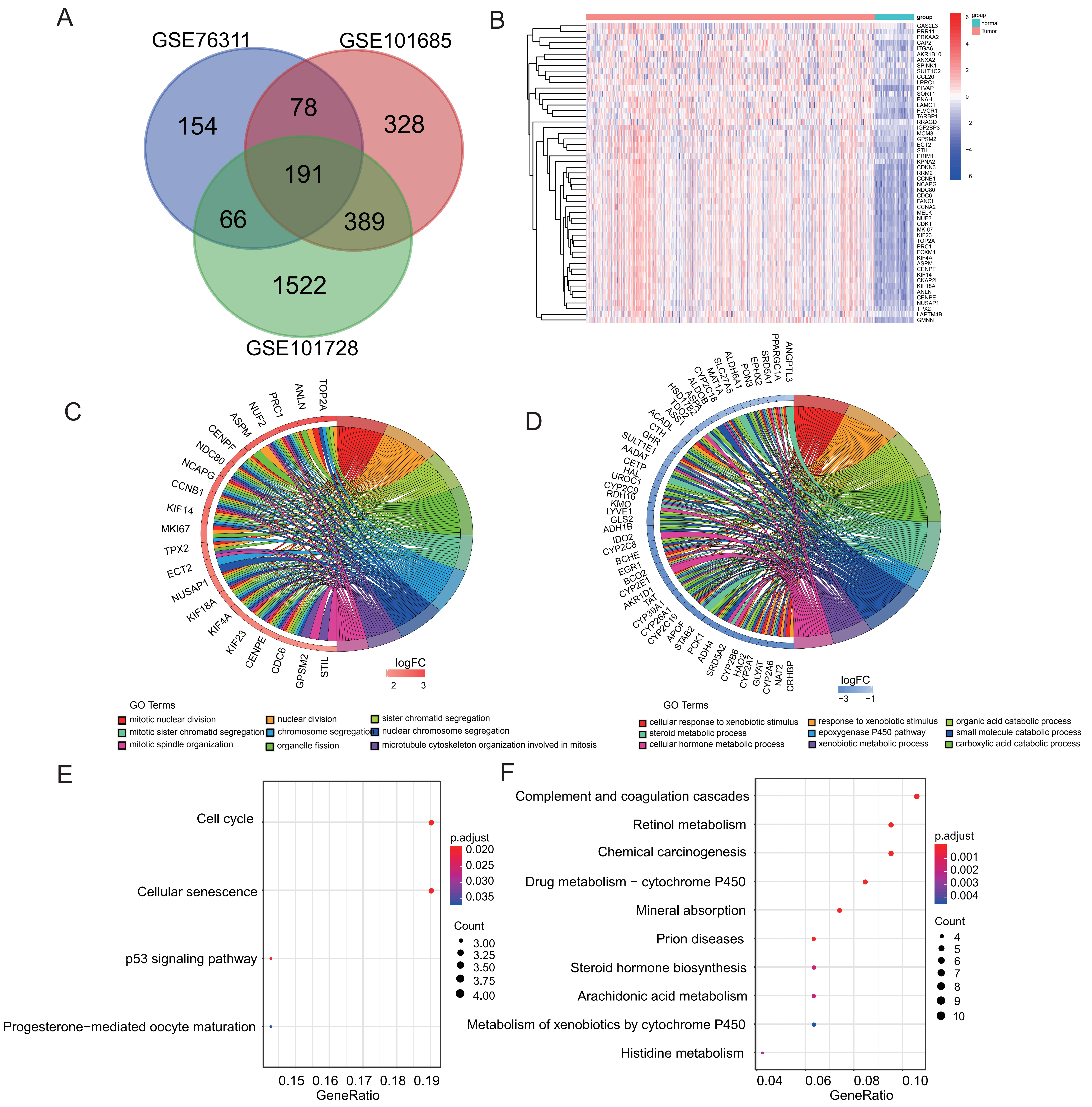
A
GSE76311
GSE101685
GSE101728
154
78
328
191
66
389
1522
B
group
normal
Tumor
C
logFC
GO Terms
mitotic nuclear division
nuclear division
sister chromatid segregation
mitotic sister chromatid segregation
chromosome segregation
nuclear chromosome segregation
mitotic spindle organization
organelle fission
microtubule cytoskeleton organization involved in mitosis
D
logFC
GO Terms
cellular response to xenobiotic stimulus
response to xenobiotic stimulus
organic acid catabolic process
steroid metabolic process
epoxygenase P450 pathway
small molecule catabolic process
cellular hormone metabolic process
xenobiotic metabolic process
carboxylic acid catabolic process
E
Cell cycle
Cellular senescence
p53 signaling pathway
Progesterone−mediated oocyte maturation
p.adjust
Count
GeneRatio
F
Complement and coagulation cascades
Retinol metabolism
Chemical carcinogenesis
Drug metabolism − cytochrome P450
Mineral absorption
Prion diseases
Steroid hormone biosynthesis
Arachidonic acid metabolism
Metabolism of xenobiotics by cytochrome P450
Histidine metabolism
p.adjust
Count
GeneRatio
